# Tracking the glossopharyngeal nerve pathway through anatomical references in cross-sectional imaging techniques: a pictorial review

**DOI:** 10.1007/s13244-018-0630-5

**Published:** 2018-06-13

**Authors:** José María García Santos, Sandra Sánchez Jiménez, Marta Tovar Pérez, Matilde Moreno Cascales, Javier Lailhacar Marty, Miguel A. Fernández-Villacañas Marín

**Affiliations:** 1 Radiology Department, University General Hospital JM Morales Meseguer, University of Murcia, Murcia, Spain; 2 Radiology Department, University General Hospital JM Universitario Morales Meseguer, C/ Marqués de los Velez s/n, 30008 Murcia, Spain; 3 Radiology Department, University Hospital Santa Lucía, University of Murcia, Cartagena (Murcia), Spain; 4 Department of Anatomy, Murcia School of Medicine, University of Murcia, Murcia, Spain; 5 Radiology Department, Hospital Barros Luco Trudeau, Santiago de Chile, Chile

**Keywords:** Anatomy, Cranial nerves, Central nervous system, Tomography, X-ray computed, Magnetic resonance imaging

## Abstract

**Abstract:**

The glossopharyngeal nerve (GPN) is a rarely considered cranial nerve in imaging interpretation, mainly because clinical signs may remain unnoticed, but also due to its complex anatomy and inconspicuousness in conventional cross-sectional imaging. In this pictorial review, we aim to conduct a comprehensive review of the GPN anatomy from its origin in the central nervous system to peripheral target organs. Because the nerve cannot be visualised with conventional imaging examinations for most of its course, we will focus on the most relevant anatomical references along the entire GPN pathway, which will be divided into the brain stem, cisternal, cranial base (to which we will add the parasympathetic pathway leaving the main trunk of the GPN at the cranial base) and cervical segments. For that purpose, we will take advantage of cadaveric slices and dissections, our own developed drawings and schemes, and computed tomography (CT) and magnetic resonance imaging (MRI) cross-sectional images from our hospital’s radiological information system and picture and archiving communication system.

**Teaching Points:**

• *The glossopharyngeal nerve is one of the most hidden cranial nerves.*

• *It conveys sensory, visceral, taste, parasympathetic and motor information.*

• *Radiologists’ knowledge must go beyond the limitations of conventional imaging techniques.*

• *The nerve’s pathway involves the brain stem, cisternal, skull base and cervical segments.*

• *Systematising anatomical references will help with nerve pathway tracking.*

## Introduction

The glossopharyngeal nerve (GPN) or IX cranial nerve is one of the most unattended cranial nerves in imaging examinations. It is not depicted for most of its course and, when affected, clinical manifestations are usually not characteristic. In fact, the GPN and vagus nerve interact so closely that an isolated GPN dysfunction might not be clinically discriminated if vagus nerve function is not impaired [1], except in the particular case of glossopharyngeal neuralgia [1–5]. When a cranial nerve injury is suspected, the nerve’s pathway must be considered from the brain stem to the target organs [3]. In a conventional magnetic resonance imaging (MRI), the vast majority of cranial nerves are generally visible only in a short segment but are affected by visible diseases. Therefore, by deepening anatomic knowledge, radiologists who interpret cross-sectional images can partially overcome that limitation.

Previous radiological publications have not been especially focused on the anatomic relationships of cranial nerves or on the GPN. Those reviews have placed more emphasis on diseases that may affect the cranial nerves [3, 4, 6–8]. For that reason, and based on an electronic poster (EPOS) presented at the 2013 European Congress of Radiology [9], the aim of this article is to review the GPN from an anatomical point of view, based on schematics, illustrations and cadaveric specimens, and to identify the anatomical references that indicate the path of the nerve in cross-sectional images. The involvement of the GPN’s pathway in pathological processes is beyond the scope of this anatomical review, and the reader can find other imaging reports elsewhere [3, 4, 6–8].

## Functional anatomy and clinical manifestations

The GPN conveys: (1) sensory afferents (retroauricular region), visceral afferents (posterior third of the tongue, pharyngeal tonsil, posterior pharynx, middle ear and Eustachian tube) and taste afferents (posterior third of the tongue); (2) parasympathetic afferents (carotid sinus baroreceptors and carotid body chemoreceptors) and efferents (parotid gland); and (3) motor efferents (stylopharyngeus muscle) [1, 2, 8–16]. It plays a role in swallowing, which involves a reflex arc that begins at the taste buds located in the posterior third of the tongue and stimulates the parotid to secrete an ideal amount of saliva fluid for swallowing [1, 16]. It also carries somatosensory information from muscles and the ear, the pharynx and tongue mucous, and information from the carotid sinus [1, 2, 8–16]. Therefore, though isolated involvement is rare, clinical manifestations of a GPN injury may be external earache, mild dysphagia and taste changes in the posterior third of the tongue; the swallowing reflex will be altered on the side of the lesion, the uvula will be deviated to the contralateral side and the sensitivity of the pharynx, palate and tongue will be affected. Changes in the quantity and quality of saliva may be present due to parotid involvement, and tachycardia carotid sinus dysfunction could be affected; finally, glossopharyngeal neuralgia, similar to trigeminal neuralgia, is an isolated GPN process that occurs very occasionally and is characterised by lancinating pain at the base of the tongue and palate [1–4].

## Imaging studies

The images retrieved and included in this manuscript were acquired in a multislice spiral computed tomography (CT), GE LightSpeed VCT 64 (Milwaukee, WI, USA) and a GE Signa HDx 1.5T MRI scanner (Milwaukee, WI, USA). All images corresponded to clinical examinations performed according to our standard cranial, skull base and cervical CT and MRI protocols (Table 1).

**Table 1:** Clinical computed tomography (CT) and magnetic resonance imaging (MRI) protocols used in the current review

CT protocol		Acquisition	Detector coverage	Pitch	Table speed	Rotation time	Kv	mA max	ASIR	Matrix	FOV
Skull base		Spiral	20 cm	0.531:1	10.62 cm/s	1 s	120	600	40%	512	25 cm
Cranial		Spiral	20 cm	0.531:1	10.62 cm/s	0.8 s	120	280	40%	512	25 cm
Neck		Spiral	20 cm	0.531:1	10.62 cm/s	0.8 s	120	280	40%	512	25 cm
MRI protocol	Sequence	TR	TE	TI	ET	BW	Matrix	FOV	ST	SP	NEX
Brain	T2W axial	5600 ms	100 ms	–	22	41.67 KHz	512 × 384	26 cm	5 mm	1 mm	2
	T2W coronal	4400 ms	102 ms	–	22	50 KHz	256 × 256	24 cm	5 mm	1 mm	2
	T2W coronal	2800 ms	102 ms	–	22	41.67 KHz	320 × 256	24 cm	3 mm	0 mm	2
Cervical	T1W sagittal	3000 ms	22 ms	Auto	9	41.67 MHz	384 × 224	26 cm	3 mm	0 mm	2
Neck	T1W axial	475 ms	10 ms	–	3	20.83 KHz	256 × 256	18 cm	4 mm	1 mm	2
	T2W axial	4200 ms	85 ms	–	13	15.63 MHz	256 × 256	18 cm	4 mm	1 mm	3
	T1W coronal	350 ms	7 ms	–	3	31.25 MHz	256 × 192	18 cm	4 mm	1 mm	1

*Kv* kilovoltage; *mA max* maximum milliamperage; *ASIR* adaptive statistical iterative reconstruction; *FOV* field of view; *TR* time of repetition; *TE* time of echo; *TI* time of inversion; *ET* echo train; *BW* bandwidth; *ST* slice thickness; *SP* spacing; *NEX* number of excitations

## Cross-sectional anatomy

For the purpose of systematisation, we will follow the pattern of Policeni and Smoker [3]. According to these authors, the path of the lower cranial nerves is divided into the brain stem, cisternal, cranial base (to which we will add the parasympathetic pathway leaving the main trunk of the GPN at the cranial base) and cervical segments, which, in this case, is practically reduced to the suprahyoid compartment [4].

### Origin in the brain stem

**Fig. 1:**
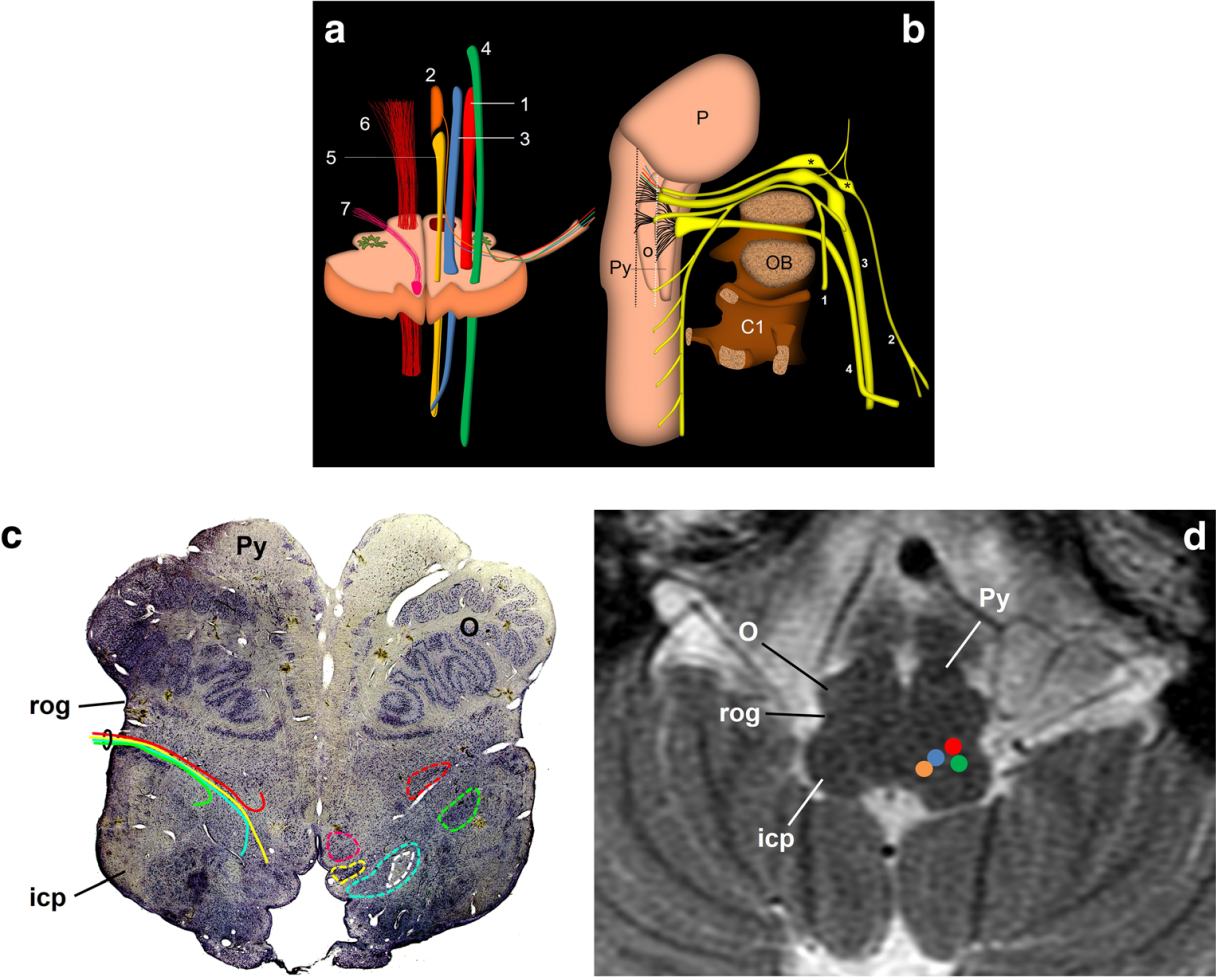
Schematic representation of the glossopharyngeal nerve. **a** Transversal section of the medulla oblongata at the level of the inferior olivary nucleus. Efferent nuclei: nucleus ambiguous (1) and inferior salivatory nucleus (2); afferent nuclei: solitary nucleus (3) and sensitive trigeminal nucleus (4). Other relevant structures: dorsal motor nucleus of the vagus nerve (5), pyramidal tract (6) and hypoglossal nerve (7). **b** Schematic drawing of the lower cranial nerves representing their mutual relationships and course from the brain stem to the cranial exit. 1: spinal nerve; 2: glossopharyngeal nerve; 3: vagus nerve; 4: hypoglossal nerve; P: pons; O: inferior olivary nucleus; Py: pyramid; C1: atlas; OB: occipital bone. The hypoglossal nerve exits the cranium through the anterior condylar canal; the glossopharyngeal, vagus and spinal nerves exit the cranium along the jugular foramen. *Glossopharyngeal nerve nodes; black dotted line: retro-olivary groove where the glossopharyngeal nerve exits the medulla oblongata with the vagus and spinal nerves. The white dotted line draws the groove between the pyramid and the inferior olivary nucleus, where the hypoglossal nerve exits the brain stem. **c**, **d** Upper medulla oblongata as viewed from below. Micrographic slice showing the glossopharyngeal nerve nuclei (**c**) and axial T2-weighted brain MRI at the same level (**d**). In the anatomical slice (Nissl stain), left nuclei are circumscribed by dotted lines. All nuclei are behind the retro-olivary groove (rog). The nerve’s pathway is represented on the right side of the sample, between the retro-olivary nucleus and the inferior cerebellar peduncle (icp). Colours as in **a** (the white dotted circle encloses the solitary tract)

The GPN and vagus nerve are mixed nerves that contain motor, branchial, sensory and autonomic fibres [16]. Both nerves have a common origin in the upper medulla oblongata and share three nuclei: the motor, the parasympathetic and the special sensory nuclei. Moreover, they convey general sensory information into the spinal trigeminal tract [1–3, 8, 11, 15–17]. Briefly, the motor nucleus involves the upper end of the nucleus ambiguous and innervates the stylopharyngeus muscle. The lower salivary nucleus sends efferent fibres to the parotid gland. General cutaneous and visceral sensory information travels through the GPN, joins the spinal trigeminal tract and ends in the spinal trigeminal nucleus. Finally, the special sensory nucleus is the solitary tract or gustatory nucleus. It receives taste sensation fibres through the solitary tract. The nucleus of the solitary tract also receives afferent impulses from the carotid sinus through the GPN [1–3, 7, 8, 10, 13, 15–17]. All nuclei are located behind the inferior olivary nucleus (Fig. 1).

### Brain stem exit and cisternal segment

**Fig. 2:**
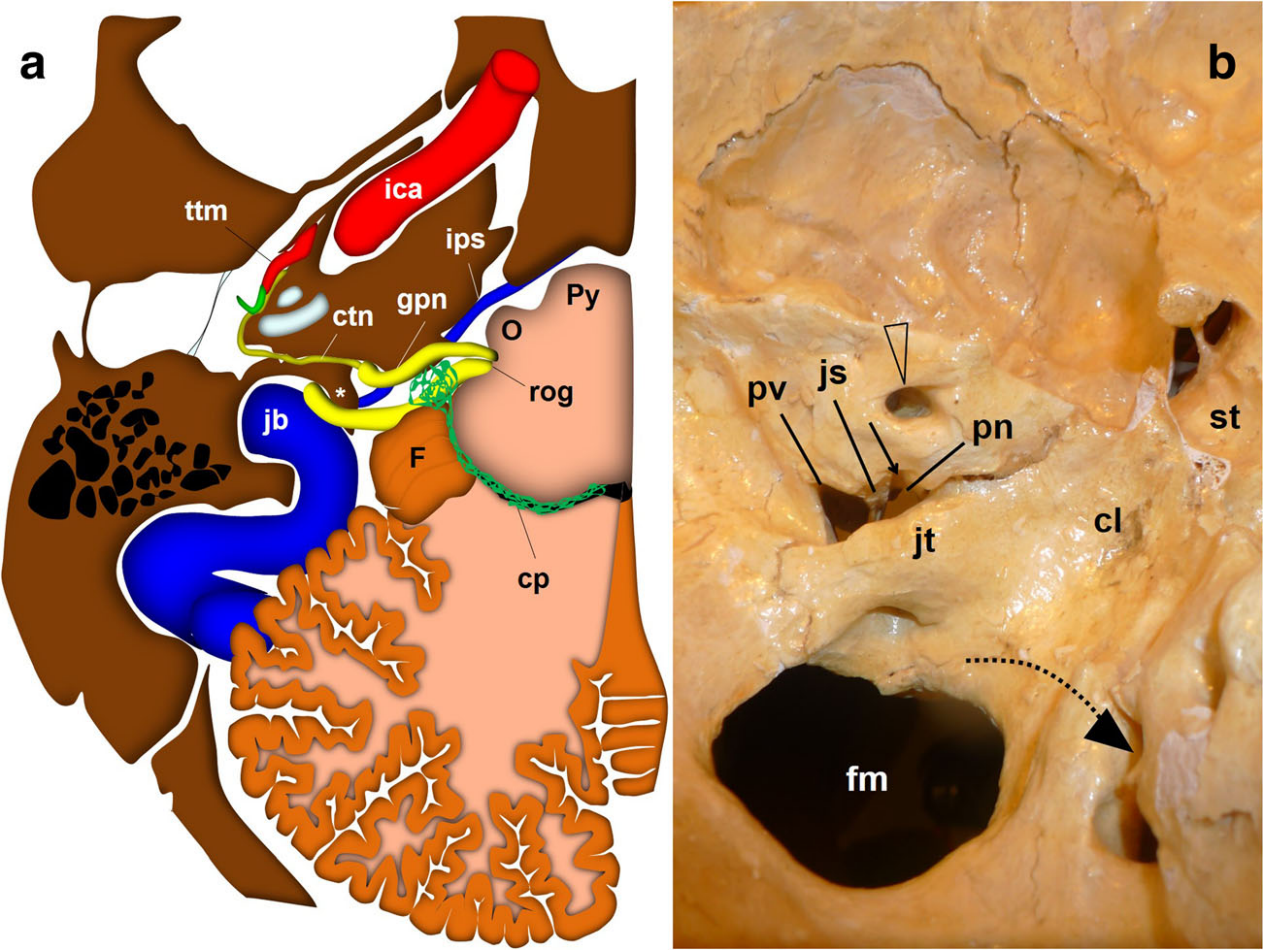
The glossopharyngeal nerve (gpn), cisternal portion. **a** Schematic drawing of a transversal slice at the level of the medulla oblongata (view from above). The gpn exits the medulla at the retro-olivary groove (rog) and crosses through the cerebellomedullary cistern to the jugular foramen. Within the cistern, it lays below and in front of the choroid plexus (cp) of the fourth ventricle and the cerebellar flocculus (F). The glossopharyngeal nerve exits the skull medial and anterior to the jugular spine (*). At that level, it is closely related to the inferior petrosal sinus (ips). The glossopharyngeal nerve crosses first over the sinus to be anterior to the sinus at the extracranial verge of the jugular foramen, posterior to the internal carotid artery. ctn: caroticotympanic or Jacobson nerve; ica: internal carotid artery; jb: jugular bulb; mo: medulla oblongata; O: inferior olivary nucleus; P: Pons; Py: pyramid; ttm: tensor tympani muscle; va: vertebral arteries. **b** Skull base, view from inside. The lower cranial nerves have a close relationship with the jugular tubercle (jt). The glossopharyngeal nerve (represented by the dotted black arrow) exits through the pars nervosa of the jugular foramen, split from the pars vascularis (pv) by the jugular spine (js). cl: clivus; fm: foramen magnum; st: sella turcica; arrowheads: internal auditory meatus

**Fig. 3:**
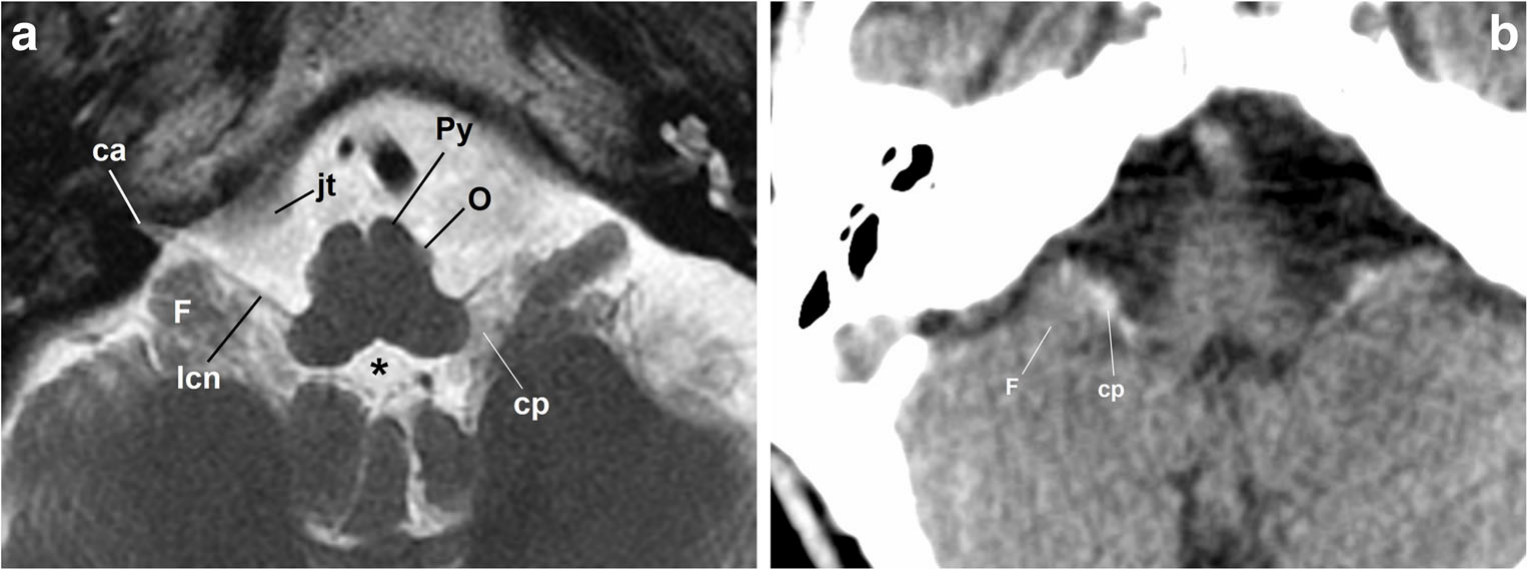
**a** Axial fast spin-echo T2-weighted brain MRI at the level of the medulla oblongata. The image shows the lower cranial nerves (lcn) passing by the flocculus (F), the choroid plexus (cp) outside the fourth ventricle (*) and the jugular tubercle (jt) as they approach the cochlear aqueduct (ca); O: inferior olivary nucleus; Py: pyramid. **b** Axial non-enhanced brain CT scan, brain window. The flocculus (F) and the choroid plexus (cp) are reliable references to determine the position of the nearby glossopharyngeal nerve

The GPN emerges in the cerebellomedullary cistern from the medulla oblongata immediately below the bulbopontine sulcus at the level of the retro-olive groove, between the inferior olivary nucleus and the inferior cerebellar peduncle [7, 8, 10] (Fig. 1). At this level, it is immediately above the vagus and spinal nerves (Fig. 2). Within the cistern, the three nerves rest on the posterior margin of the jugular tubercle of the occipital bone (Figs. 2 and 3), crossing laterally close to the anterior and inferior margin of the flocculus and the choroid plexus protruding from the fourth ventricle (Fig. 3). When it arrives at the skull base, the GPN enters an exclusive cranial exit near the top of the jugular foramen [10, 13, 14] (Fig. 2). The cranial nerves are visible in the cistern with MRI [4, 15, 18, 19] but not with CT, so the cerebellar flocculus, the choroid plexus and the jugular tubercle become critical anatomical references.

### The jugular foramen and parasympathetic segment

**Fig. 4:**
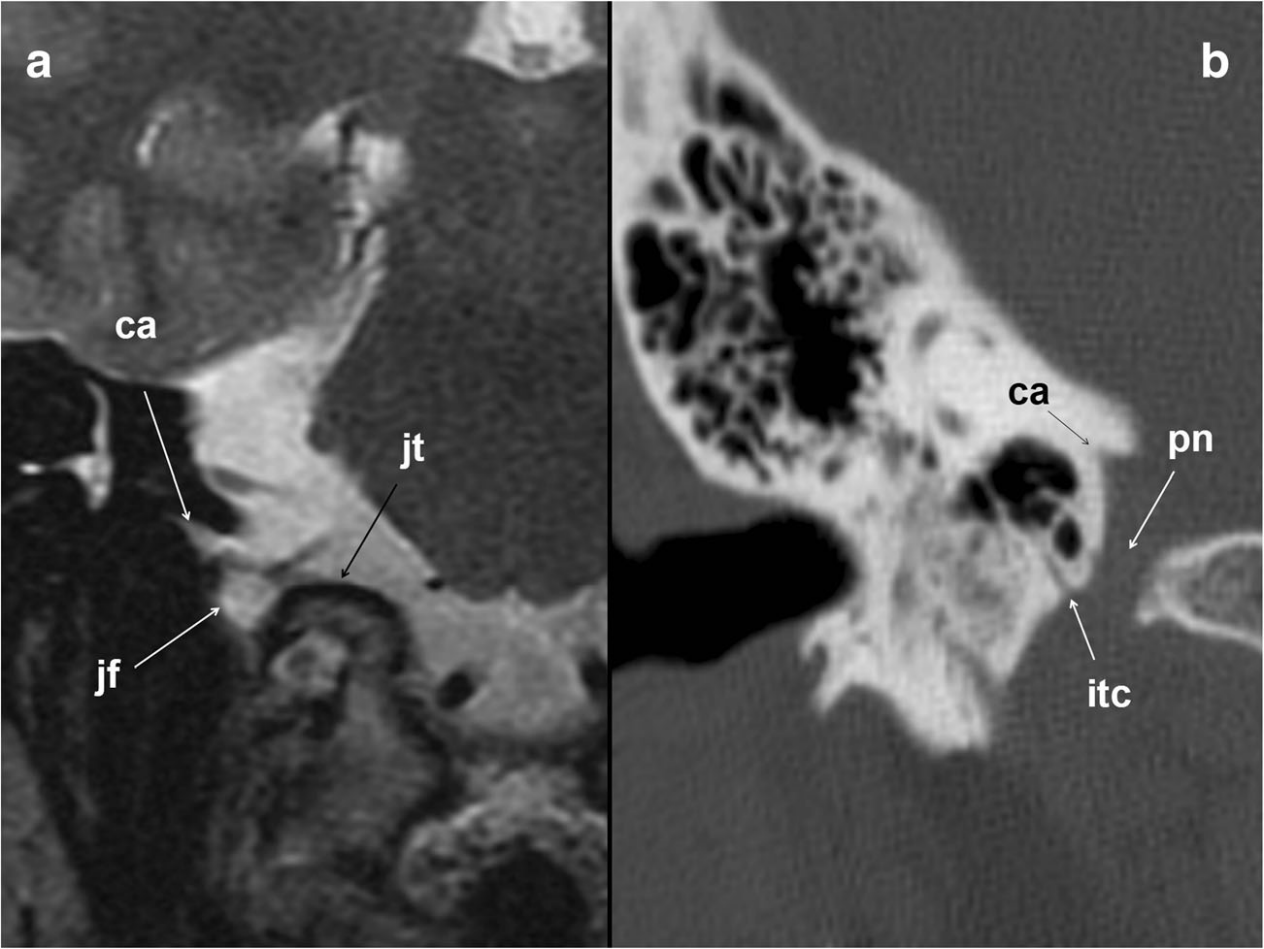
The glossopharyngeal nerve at the jugular foramen. **a** Coronal T2-weighted brain MRI at the level of the jugular foramen (jf). The image shows the relationship between the cochlear aqueduct (ca) above, and the jugular foramen and jugular tubercle (jt) below. **b** Skull base CT scan; coronal reconstruction of the right temporal bone, bone window. The image shows the inferior tympanic canaliculus (itc) for the inferior tympanic or Jacobson’s nerve at the jugular spine. ca: cochlear aqueduct; pn: pars nervosa

**Fig. 5:**
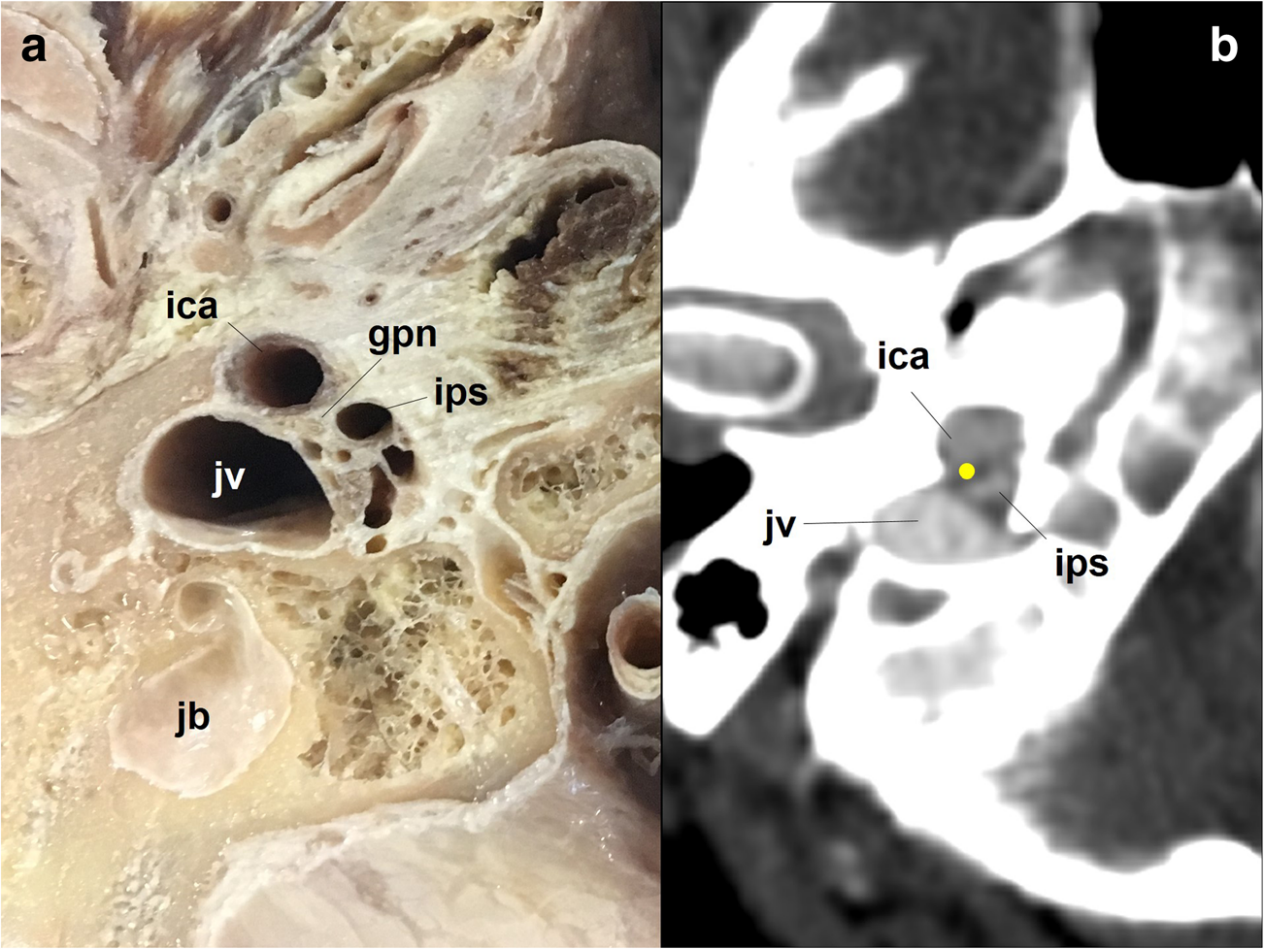
The glossopharyngeal nerve (gpn) at the jugular foramen. **a** Cadaveric section at the level of the skull base; view from below. The glossopharyngeal nerve exits the skull close to the inferior petrosal sinus (ips), the internal carotid artery (ica) and the jugular vein (jv). jb: jugular bulb. **b** Corresponding axial neck CT scan at the extracranial verge of the jugular foramen; right temporal bone, soft tissues window. The glossopharyngeal nerve is represented with a yellow dot

The cochlear aqueduct, opening just above the GPN entrance in the jugular foramen [14, 15, 19–21], is the first anatomical reference (Figs. 3 and 4). In the jugular foramen, the GPN runs through the anteromedial portion or pars nervosa (petrosal fossula), separated from the posterolateral portion or pars vascularis (exit for the vagus and spinal nerves and the internal jugular vein) by the jugular spine (Fig. 2) and a fibrous septum (petro-occipital ligament), which is sometimes ossified [3, 6, 8, 13, 14]. At the entrance to the foramen, the GPN, vagus and spinal nerves are initially arranged in an obliquely posteroanterior-lateromedial distribution, between the jugular bulb (posterolateral) and the inferior petrosal sinus (anteromedial) [21] (Fig. 2). Once at the exit of the jugular foramen, the GPN is generally in front of the inferior petrosal sinus before draining into the internal jugular vein [6, 20, 21] (Figs. 2 and 5). Still within the foramen, the GPN shows two focal expansions or nodes [11] (Fig. 1), which are normally not visible in sectional high-resolution clinical images at 1.5T MRI [18] but have been observed with 3T MRI [21]. The superior node conveys general sensitive information and is located next to the opening of the cochlear aqueduct [21]. The lower node (Andersch node) handles the visceral sensory, gustatory and carotid [6, 16] innervations, and is located approximately 3 mm below [21].

**Fig. 6:**
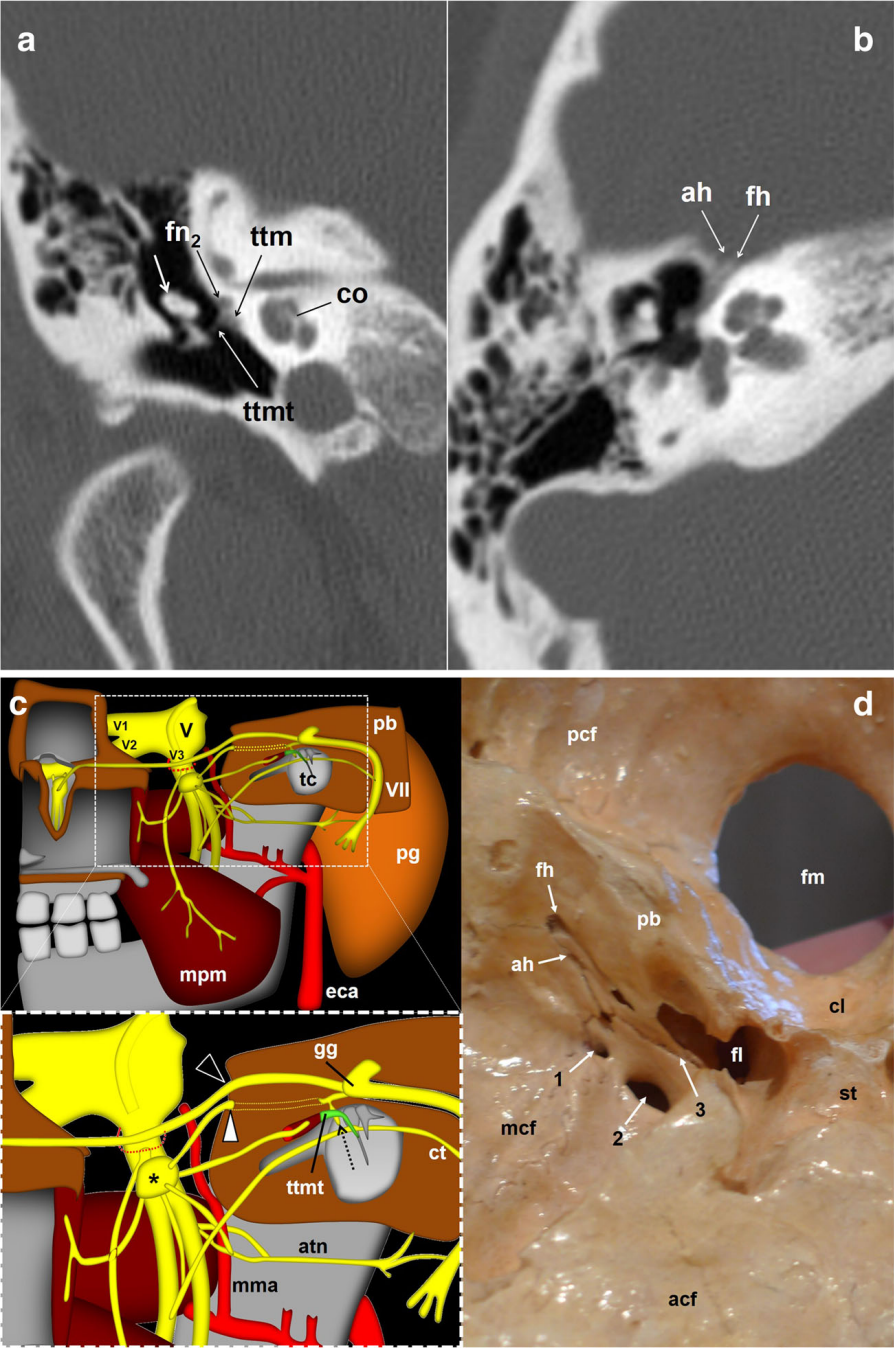
The glossopharyngeal nerve (gpn) and the tympanic nerve of Jacobson. **a** Skull base CT scan; coronal reconstruction of the right temporal bone, bone window. Once arriving at the tympanic cavity at the level of the promontory (p), the tympanic nerve forms a submucous plexus within the middle ear including the mastoid antrum and Eustachian tube. At the level of the tendon (ttmt) of the tensor tympani muscle (ttm), a branch of the tympanic plexus exits the cavity through a small duct and courses forward next to the facial nerve (fn_2_); short white arrow: malleus; co: cochlea. **b** Skull base axial CT scan; right temporal bone, bone window. The glossopharyngeal nerve fibres will exit the petrosal bone through the accessory hiatus (ah) after joining the lesser petrosal nerve of the facial nerve. fh: fallopian hiatus. **c** Schematic drawing of the right infratemporal fossa, view from inside. Glossopharyngeal nerve relationships with the facial (VII) and trigeminal (V) nerves. The lower drawing represents a detailed view of the region of interest (dashed rectangle). The deep great petrosal nerve (dotted arrow) comes up from the tympanic plexus at the level of the tendon of the tensor tympani muscle (ttmt) to merge with the lesser superficial pretrosal nerve coming from the geniculate ganglion (gg) of the facial nerve. The resulting nerve exits the petrosal bone (pb) to the middle cranial fossa at the level of the accessory hiatus (white arrowhead). The hiatus is located close and lateral to the fallopian hiatus (black arrowhead), through which the greater petrosal nerve enters into the skull. atn: auriculotemporal nerve; ct: chorda tympani; eca: external carotid artery; mpm: medial pterygoid muscle; mma: middle meningeal artery; pg: parotid gland; tc: tympanic cavity; V1: first trigeminal nerve branch; V2: second trigeminal nerve branch; V3: third trigeminal nerve branch; *Otic ganglion; dashed red circle: foramen ovale. **d** Skull base viewed from inside. The image shows the fallopian hiatus (fh) and the accessory hiatus (ah) on the right side, as well as the foramen spinosum (1), foramen ovale (2) and sphenopetrosal fissure (3), through which the lesser petrosal nerve that contains the glossopharyngeal nerve fibres may exit the cranium. acf: anterior cranial fossa; cl: clivus; fl: foramen lacerum; fm: foramen magnum; mcf: middle cranial fossa; pcf: posterior cranial fossa; st: sella turcica

The tympanic nerve or Jacobson nerve leaves the GPN from the lower node [10–14]. Fibres of the GPN course through this nerve and have a long cranial and parapharyngeal path, jumping from cranial nerve VII (the facial nerve) to cranial nerve V (the trigeminal nerve), and targeting the parotid gland finally. The first anatomical reference for the Jacobson nerve is the inlet of the lower tympanic canaliculus in the jugular spine, which enters just when leaving the GPN [3, 6] and through which it reaches the medial wall of the tympanic cavity at the level of the cochlear promontory [7, 14, 22] (Figs. 2, 4 and 6). Inside the tympanic cavity, the nerve forms a submucosal plexus that conveys sensitive information from the middle ear mucosa, antrum, mastoid air cells and Eustachian tube [3, 8, 10, 13–15]. At the level of the tendon of the tensor tympani muscle, it gives off a small nerve branch (deep great petrosal nerve) that will bind the lesser superficial petrosal nerve coming from the geniculate ganglion of the facial nerve [6, 11, 12, 23]. The course of the resultant nerve (the lesser petrosal nerve) can be followed by identifying the geniculate ganglion and the ducts in front, until the nerve enters the middle cranial fossa through the accessory hiatus, lateral to the exit of the greater petrosal nerve, which exits through the fallopian hiatus [10–12] (Fig. 6).

**Fig. 7:**
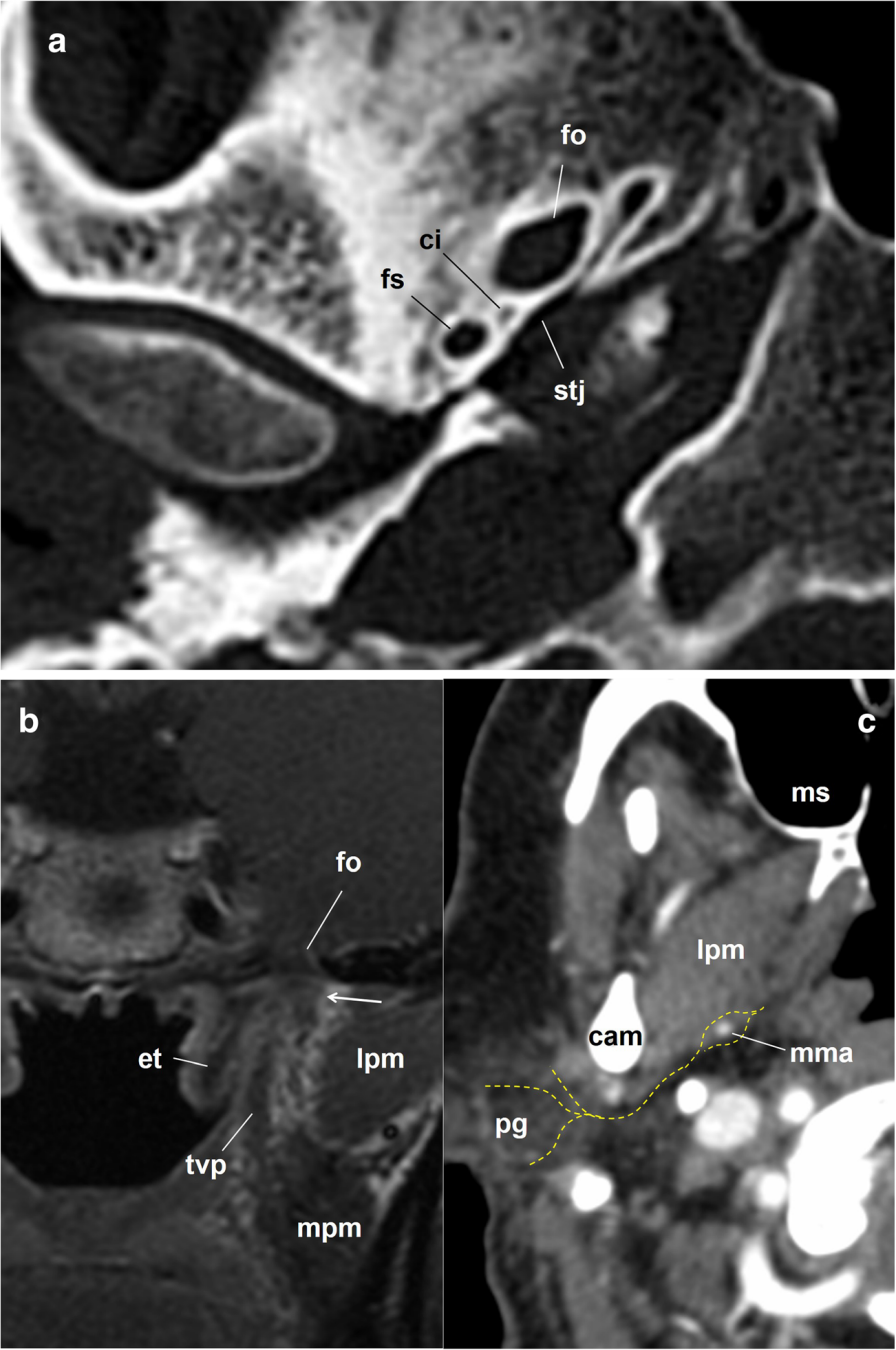
The glossopharyngeal nerve. **a** Skull base axial CT scan; right temporal bone, bone window. The image shows the foramen ovale, the foramen spinosum, the canaliculus innominatus (ci) of Arnold and the spheno-temporal junction, all of which are possible exit pathways of the lesser petrosal nerve. **b** Coronal fast spin-echo contrast-enhanced T1-weighted brain MRI, left side skull base. The images show the relationship between the foramen ovale (fo) and the V_3_ mandibular nerve and otic ganglion complex (arrow). et: Eustachian tube; lpm: lateral pterygoid muscle; mpm: medial pterygoid muscle; tvp: tensor veli palatini muscle. **c** Axial contrast-enhanced CT scan of the neck, soft tissues window. Glossopharyngeal nerve and auriculotemporal nerve. Just below the foramen ovale, the glossopharyngeal nerve fibres leave the otic ganglion to merge with the auriculotemporal nerve, a branch of the trigeminal nerve (the nerve pathway is represented by dashed yellow lines). Then, the nerve travels posteriorly in the parapharyngeal space to embrace the middle meningeal artery (mma), crosses along the medial edge of the condylar apophysis of the mandible (cam) and enters the parotid gland (pg). lpm: lateral pterygoid muscle; ms: maxillary sinus

Once in the middle cranial fossa, it crosses forward and medially to leave the skull variably through different exits: the foramen spinosum, foramen ovale, innominate canaliculus (located between these two foramen) or the sphenopetrosal junction [11, 23] (Figs. 6 and 7). Immediately below the skull, the GPN fibres synapse in the otic ganglion, medial to the mandibular nerve and just under the foramen ovale [10–13] (Fig. 7). The GPN postganglionic fibres leave the otic ganglion through the auriculotemporal branch of the trigeminal nerve. Now, the reference is the line that connects the mandibular nerve with the middle meningeal artery. The auriculotemporal nerve embraces the artery and then goes back through the parapharyngeal space medial to the lateral pterygoid muscle first and the neck of the mandibular condyle later, to reach the deep lobe of the parotid gland, providing it with parasympathetic innervation [8, 10, 11, 14, 22] (Fig. 7).

### Cervical segment

**Fig. 8:**
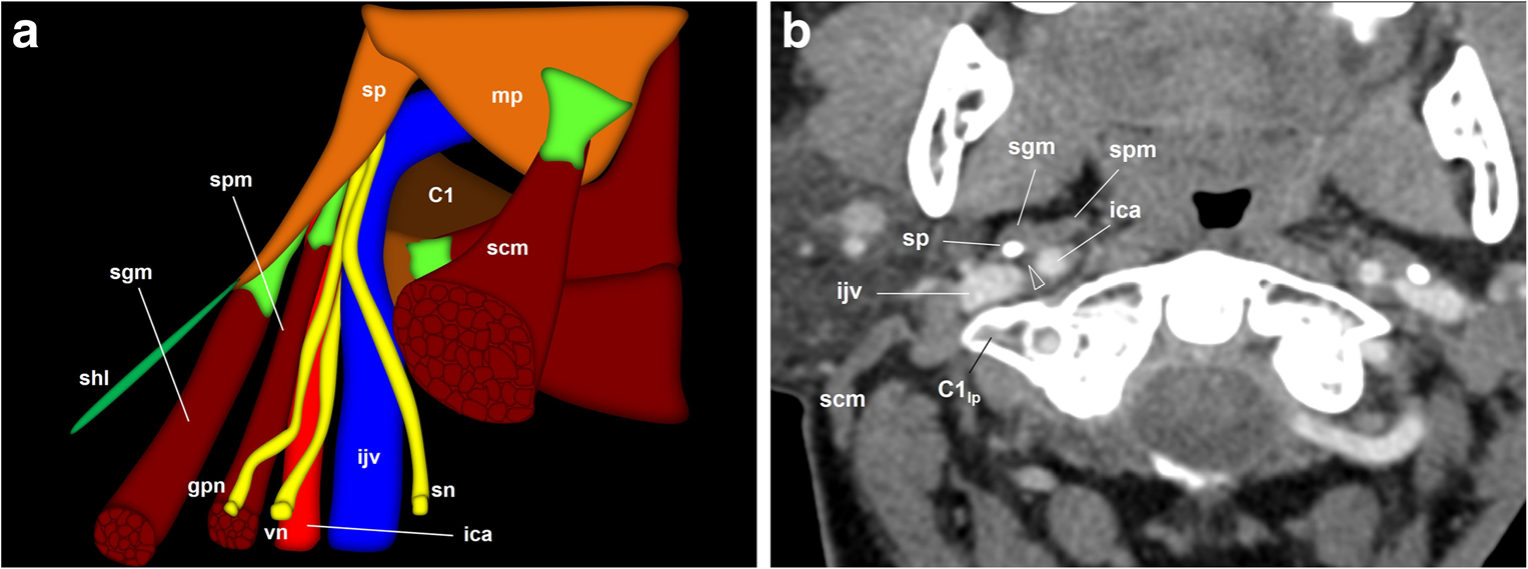
The glossopharyngeal nerve (gpn), cervical portion. **a** Schematic drawing of the glossopharyngeal nerve when leaving the left jugular foramen. The glossopharyngeal nerve is located medial to the internal jugular vein (ijv) and behind the internal carotid artery (ica) in the carotid space. The styloid process (sp) is the most lateral reference at this point. At the level of the first cervical vertebra (C1), the glossopharyngeal nerve surrounds the artery laterally and descends between the styloid process and stylopharyngeus muscle (spm). scm: sternocleidomastoid muscle; sgm: styloglossus muscle; shl: stylohyoid ligament; mp: mastoid process: sn: spinal nerve; vn: vagus nerve. **b** Axial contrast-enhanced CT scan of the neck, right side, at the level of the first cervical vertebra lateral process (C1_lp_); soft tissues window. At this point, the glossopharyngeal nerve (arrowhead) is located between the internal carotid artery anteriorly and the internal jugular vein behind. The styloid process is now in front of the glossopharyngeal nerve as it turns around the lateral side of the internal carotid artery to reach the dorsal aspect of the stylopharyngeus muscle. At this point, Hering’s nerve leaves the glossopharyngeal nerve to go down to the carotid body along the anterior aspect of the internal carotid artery

**Fig. 9:**
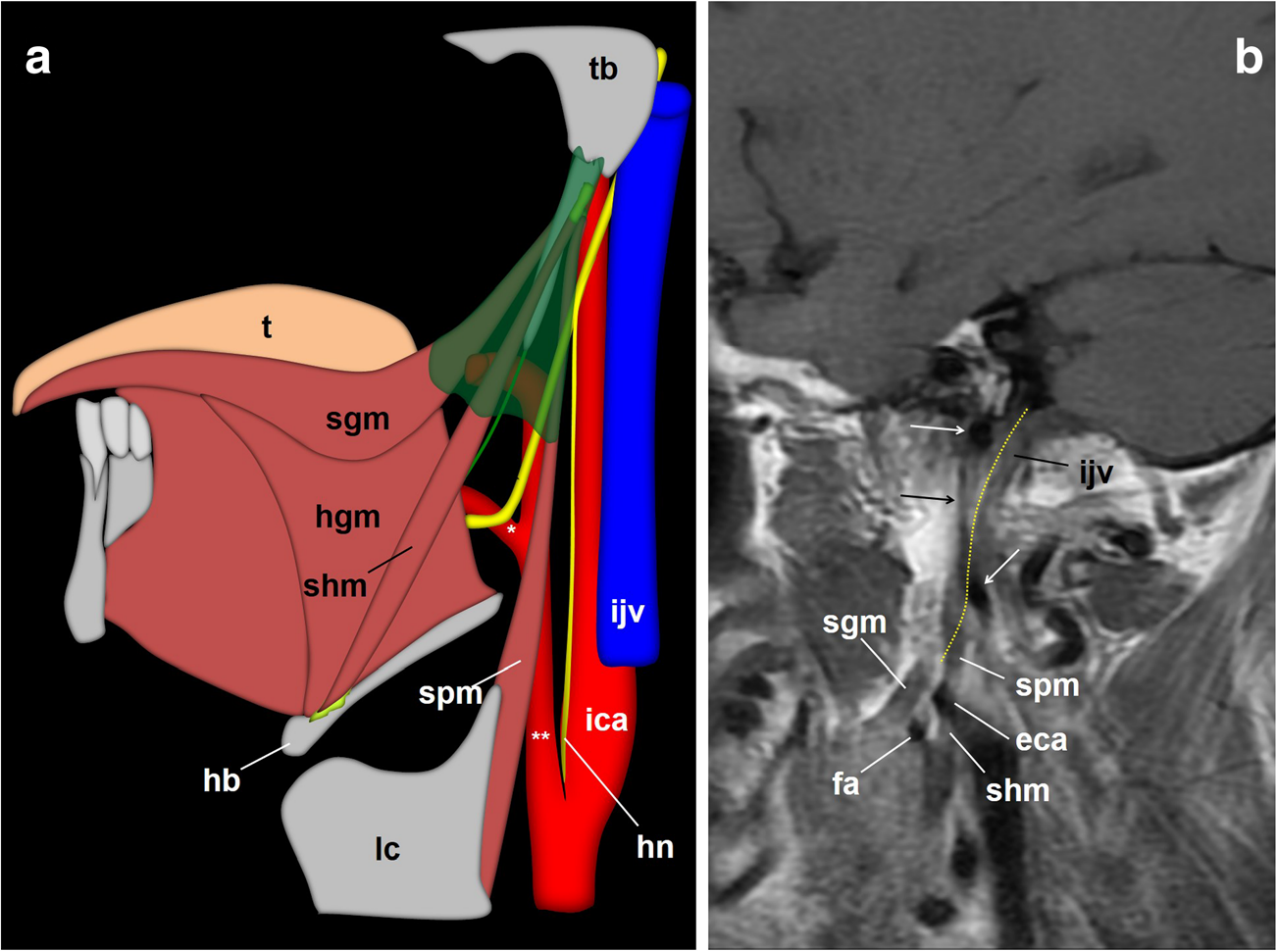
The glossopharyngeal nerve, cervical portion. **a** Schematic drawings that depict the glossopharyngeal nerve at the styloid pyramid and its oropharynx end. Once the Hering’s nerve (hn) has left the IX cranial nerve, the glossopharyngeal nerve enters a muscular tripod that consists of three styloid muscles and a fascia (shaded in green): the styloid pyramid. Within the pyramid, the glossopharyngeal nerve (arrowhead) usually runs in a triangular plane limited by the facial artery (*), the external carotid artery (**) and the styloglossus muscle (sgm). When the stylopharyngeus muscle merges with the constrictor muscles, the nerve enters the oropharynx and, finally, courses deep to the hyoglossus muscle (hgm). hb: hyoid bone; lc: laryngeal cartilage; shm: stylohyoid muscle; t: tongue. **b** Sagittal fast flair T1-weighted MRI centred at the skull base. Arrows: internal carotid artery; eca: external carotid artery; fa: facial artery; the dotted yellow line represents the expected pathway of the glossopharyngeal nerve

Immediately after leaving the jugular foramen, the GPN is located medial to the internal jugular vein and behind the internal carotid artery (Figs. 5 and 8). Now it is located in the retro-styloid or carotid space [4, 24]. When proceeding down the neck, the GPN initially has the same vascular relationships. The styloid process, the most lateral reference at this level, is also a useful mark [10, 12]. At the level of the C1 transverse process, the nerve goes around the carotid artery laterally and descends behind the styloid process and the stylopharyngeus muscle [14] (Fig. 8). When it crosses between the internal carotid artery and the internal jugular vein, the GPN gives off the carotid sinus and carotid body nerve (Hering’s nerve), which descends along the anterior wall of the internal carotid artery [11] (Fig. 9).

**Fig. 10:**
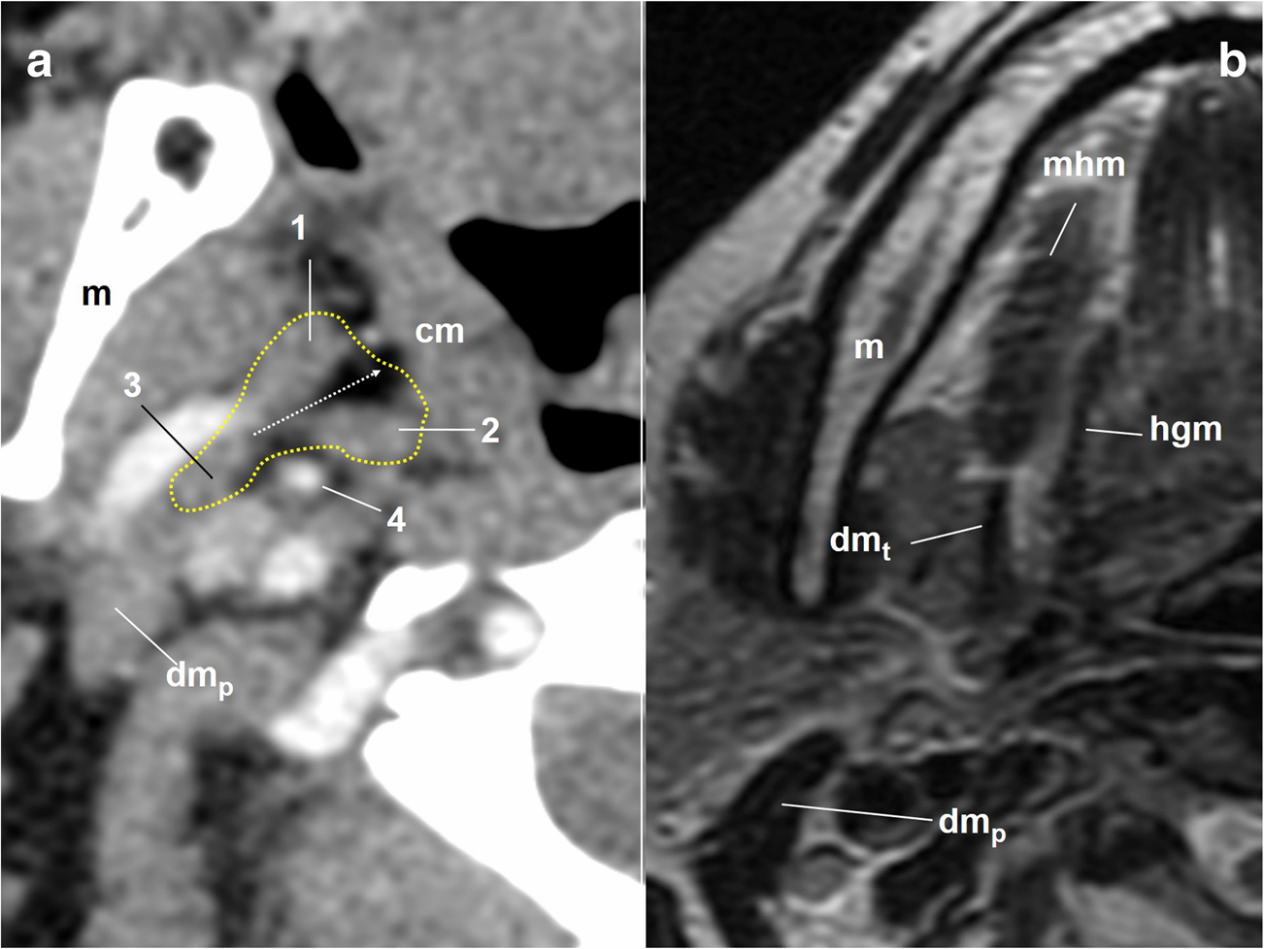
Cervical portion of the glossopharyngeal nerve. **a** Axial contrast-enhanced CT scan of the neck, right side, soft tissues window. The dotted yellow line draws the limits of the styloid pyramid. 1: styloglossus muscle; 2: stylopharyngeus muscle; 3: stylohyoid muscle; 4: external carotid artery; cm: constrictor muscles. The dotted white arrow represents the long axis of the styloid pyramid as a reference of the gpn pathway. dm_p_: posterior belly of the digastric muscle; m: mandible. **b** Axial T2-weighted MRI of the neck, right side, at the level of the intermediate tendon of the digastric muscle (dm_t_). The slice over the level of the tendon is a good reference for the entry point of the glossopharyngeal nerve in the pharynx. mhm: mylohyoid muscle

Once the GPN is anterior to the vascular structures, it leaves the close relationship with the internal carotid artery approximately at the level of the soft palate [24] and runs behind the styloid muscles (stylohyoid, styloglossus, stylopharyngeus) (Fig. 10). These muscles form a tripod that will be the reference until the nerve enters the oropharynx, especially the medial (stylopharyngeus) and the anterior (styloglossus) muscles. The styloid muscles are surrounded by a fibrous fascia (styloid diaphragm); muscles and fascia form the styloid pyramid [14] (Fig. 9). The GPN surrounds the external side of the stylopharyngeus muscle to reach its anterior surface inside the pyramid [11]. In this course, the GPN provides motor innervation to the muscle [14, 25, 26]. Once within the pyramid, the most common position of the GPN is in a triangle, in which the posterior margin is the external carotid artery, the lower one is the facial artery and the anterior one is the styloglossus muscle [27] (Fig. 9). However, this reference appears not to be suitable for the common axial slices, in which the long axis of the styloid pyramid area might be an easier anatomical key point (Fig. 10).

At approximately the point where the stylopharyngeus muscle merges with the constrictor muscles, the GPN enters the pharynx between the upper and middle constrictors [14]. At this point, the stylopharyngeus and hyoglossus muscles separate the hypoglossal nerve (lateral) from the GPN (medial). This circumstance occurs immediately above the level of the intermediate tendon of the digastric muscle [14] (Fig. 10).

Once in the pharynx, the GPN splits into pharyngeal branches, which contribute to the pharyngeal plexus of the vagus, and the lingual branch [4, 6, 10, 14, 25]. The references of the lingual branch are the lower edge of the palatine tonsil (palatoglossus and styloglossus muscles) and the hyoglossus muscle. The lingual branch reaches the tongue medial to the hyoglossus muscle to innervate the posterior third of the tongue [6, 11] (Fig. 10).

**Fig. 11:**
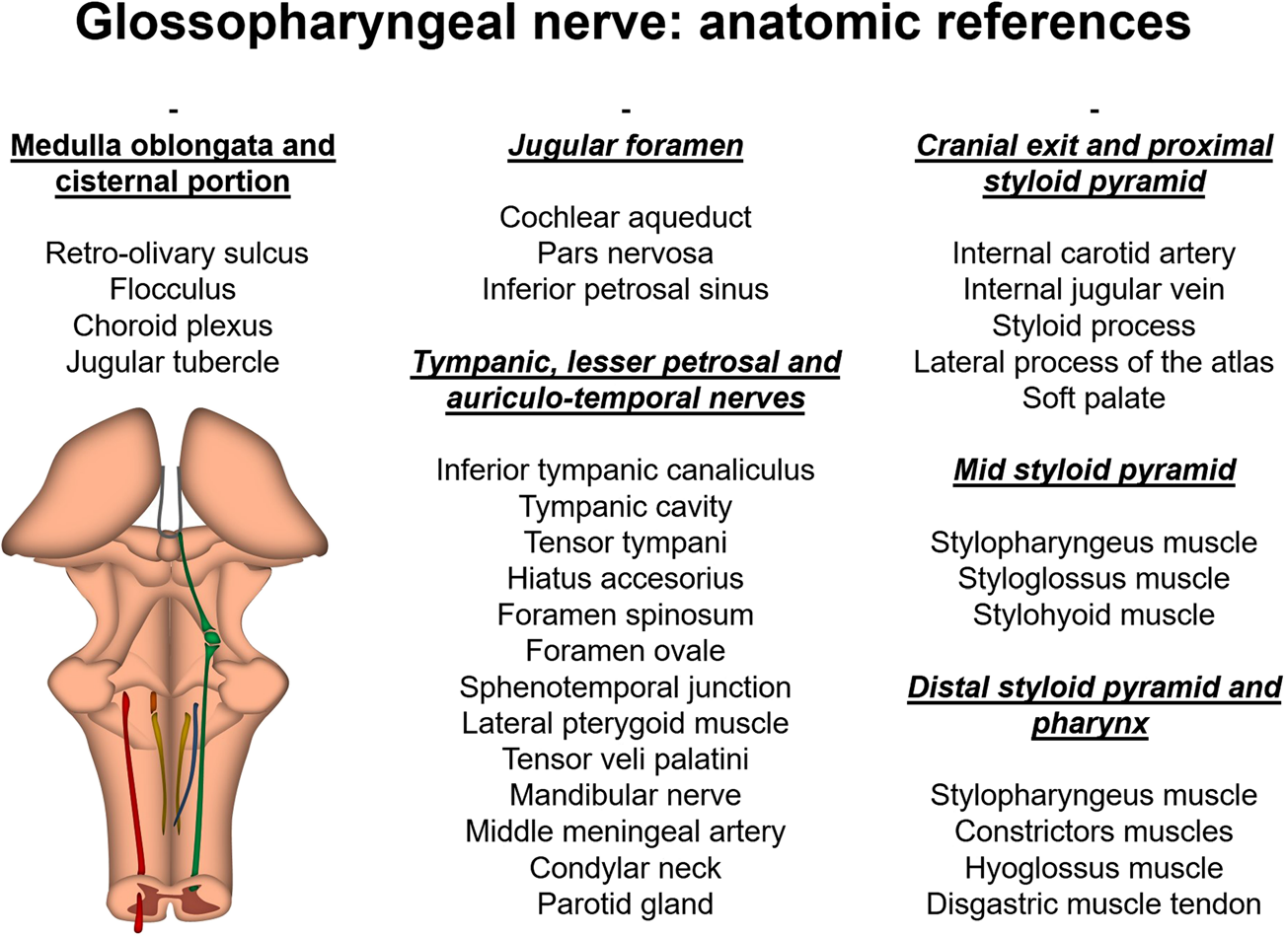
Summary of the anatomic references of the glossopharyngeal nerve in the cross-sectional conventional clinical images

All anatomic references are summarised in Fig. 11.

## Glossopharyngeal nerve dysfunction: denervation signs

**Fig. 12:**
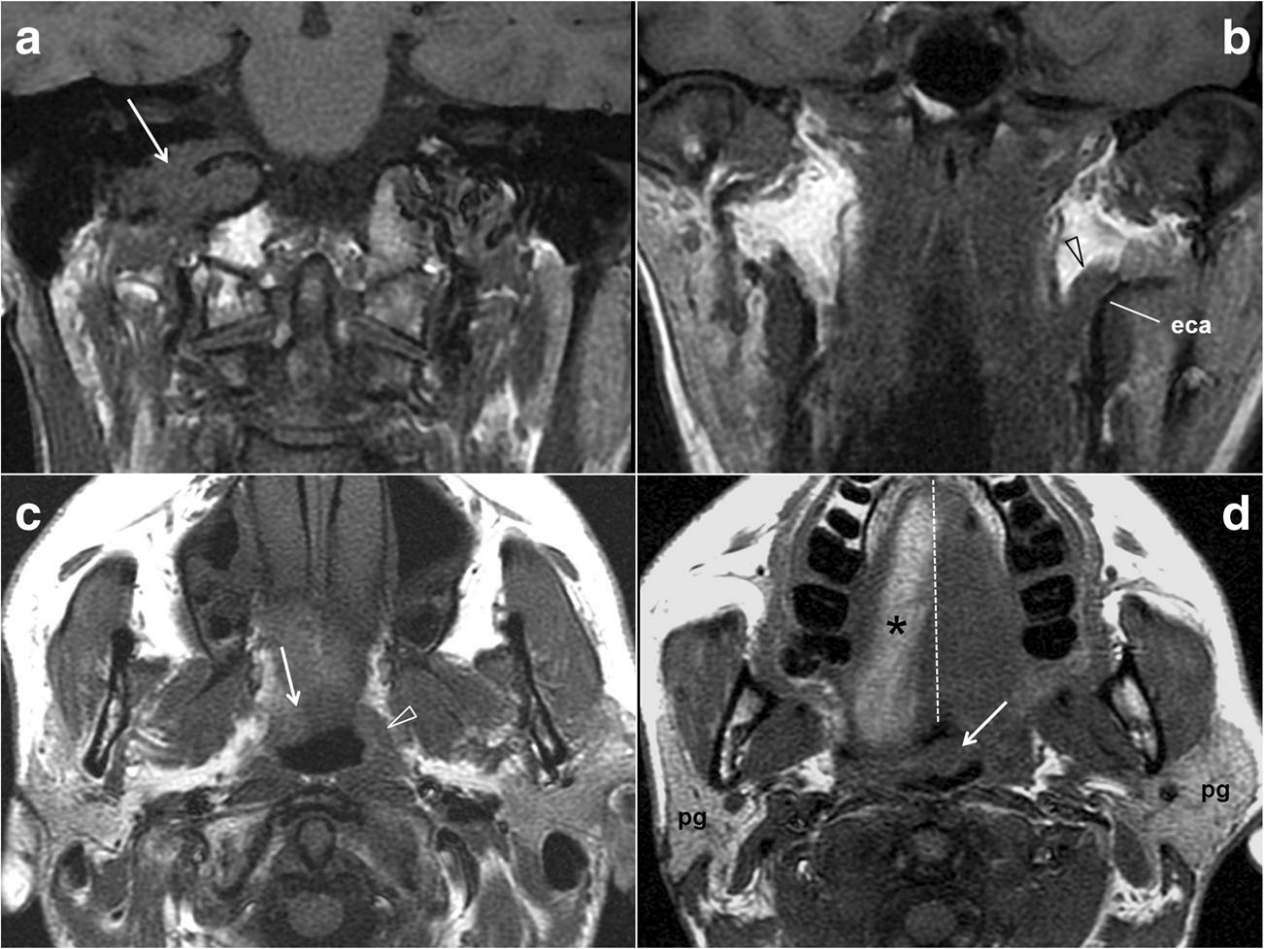
Glossopharyngeal nerve dysfunction. Low cranial nerves palsy. **a** Coronal fast spin-echo T1-weighted MRI of the neck. A slightly hypointense mass eroding the right jugular foramen (arrow) was demonstrated to be a glomus jugulare paraganglioma. **b** Coronal fast spin-echo T1-weighted MRI of the neck. The normal stylopharyngeus muscle (arrowhead) on the left and its relationship with the external carotid artery (eca) help to recognise the muscle atrophy on the right side. Stylopharyngeus muscle atrophy is one of the few specific imaging signs of glossopharyngeal nerve dysfunction. **c** Axial fast spin-echo T1-weighted MRI of the neck. An asymmetric oropharyngeal lumen due to a descending soft palate on the right (arrow) and constrictor muscle atrophy (the arrowhead points to the normal muscles on the left). These are signs of vagus nerve dysfunction, which is normally associated with glossopharyngeal nerve changes. **d** Axial fast spin-echo T1-weighted MRI of the neck. The uvula (arrow) is displaced to the left side (the dotted midline has been drawn to emphasise that displacement), which is the other typical sign of vagus nerve palsy. Intriguingly, this patient also showed a right parotid gland (pg) atrophy, which might be related to the glossopharyngeal nerve dysfunction. Also note the fat replacement of the right side of the tongue due to hypoglossal nerve palsy

Muscle denervation can be the most visible sign of neural injury, especially in larger muscles [5, 28, 29]. However, the GPN only innervates the small stylopharyngeus muscle, for which no cases of atrophy have been reported [5, 28]. It also contributes to the pharyngeal plexus of the vagus nerve, which supplies the palatoglossus and palatopharyngeus muscles, the middle and upper constrictors, and the levator muscle of the soft palate [5]. Therefore, secondary changes in cross-sectional images due to GPN denervation cannot be separated from those caused by vagus nerve injuries (Fig. 11). Imaging features are characterised by the soft palate descent at the side of the lesion, a deviated uvula to the opposite side and muscle asymmetries at the level of the torus tubarius and constrictor muscles [1, 26, 28]. Functional parotid gland changes may be unnoticed, as the other salivary glands remain unaffected [1]. More debatable is the possibility of secondary gland atrophy after denervation. Parotid atrophy has been reported in cases of chronic trigeminal denervation, as the auriculotemporal nerve conveys the gland innervations [30]. Though we found no previous articles on parotid gland atrophy related to lesions affecting the GPN pathway, tympanic neurectomies were reported to show that atrophy [31] and the radiologist should consider this possibility (Fig. 12).

## Conclusion

The novelty of this article is the specific focus on the glossopharyngeal nerve (GPN) in cross-sectional imaging, which systematises the anatomical relationships of the main trunk and its branches. It will help radiologists obtain insights into a cranial nerve that, unlike many of its counterparts, remains beyond the observer’s attention due to its inconspicuous clinical signs and scarce visibility in normal and pathological clinical images.
